# Population Challenges for Bangladesh in the Coming Decades

**Published:** 2008-09

**Authors:** Peter Kim Streatfield, Zunaid Ahsan Karar

**Affiliations:** Health and Demographic Surveillance Unit, Public Health Sciences Division, ICDDR, B, GPO Box 128, Dhaka 1000, Bangladesh

**Keywords:** Ageing, Age factor, Child survival, Climate change, Family planning, Fertility, Population growth, Urbanization, Bangladesh

## Abstract

Bangladesh currently has a population approaching 150 million and will add another 100 million before stabilizing, unless fertility can soon drop below replacement level. This level of fertility decline will require a change in marriage patterns, which have been minimal so far, even with increasing female schooling. It would also benefit from a long-awaited shift to long-term contraception. In addition to the consequence of huge population size, the density of population is already five times that of any other ‘mega’ country (>100 million), a very challenging situation for an agricultural society. Most of the future growth will be urban, increasingly in slums. Numbers of young people will not increase, but numbers of older people will increase 10-fold this century, creating a large burden on the health system, especially for chronic illnesses. High density of population means that agricultural land is virtually saturated, with very limited capacity to expand food production. Climate change may have dramatic impacts on agriculture, through flooding and drought resulting from weather changes and geopolitical influences on transborder rivers. Rising sea-levels and consequent salinity will affect crops and require shifts to alternative land use. Serious long-term planning is needed for meeting the growing needs of the population, both for distribution and consumption.

## CURRENT POPULATION SITUATION AND FUTURE GROWTH

The Bangladesh population in mid-2007 was around 147 million. The precise figure depends on assumptions about rates of growth since the adjusted census figure of 129,247,233 for 22 January 2001. The Bangladesh Bureau of Statistics (BBS) indicates an average rate of natural increase of 1.5% annually since the census. However, these figures from the BBS are almost certainly under-estimates of crude birth rate (CBR) (20-21/1,000) and crude death rate (CDR) (5-6/1,000). The BBS rates would project a population of just over 141 million whereas the Population Reference Bureau (PRB) rate of natural growth of 1.9% per annum is more likely—based on assumed CBR of 27/1,000 and CDR of 8/1,000, resulting in a projected population of 147 million.

### Population projections

**Fig. 1:**
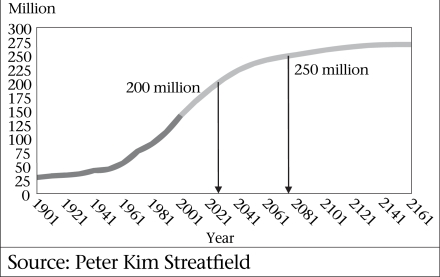
Population of Bangladesh: 10-fold growth in two centuries

A more important question is what the future popu- lation growth will be. Until the United Nations Population Division (UN PD) released the 2004 revision of population projections for all the countries of the world, there was a general agreement that Bangladesh would reach 218 million by 2050 and finally stabilize at around 260 million in mid-next century (1). However, the 2004 revision by the UN PD proposes a pessimistic upgrading of the 2050 figure to 243 million as a consequence of the decade-long fertility plateau (1993–2002). This seems excessively high. The PRB estimate is 231 million for mid-century, which is more realistic, although a new set of Bangladesh population projections should be produced, taking into account the recent demographic events. Whatever the details, it can be seen in Figure 1 that Bangladesh is only halfway along the S-curve of the ‘demographic transition’ from a historically-stable population size of around 25 million to a final size of 10-fold larger.

**Key messages**

- The density of the Bangladesh population is much higher than any other mega country.
- In 2007, Bangladesh was only halfway up the population growth curve and will reach more than 250 million later this century.
- Most of the increased population will be urban, and much of Bangladesh will essentially become a city state.
- The year that Bangladesh reaches replacement fertility is not as critical lowering fertility to less than replacement. Other Asian countries have accomplished this.
- The number of children born each year is now stabilized at slightly less than 4 million.
- Age of marriage has not increased in Bangladesh as it has in other Asian countries.
- The elderly (>60 years) will grow in population from 7 to 65 million this century, and this will make many demands and different demands on the health system.
- There has been a major reduction in overall numbers of child deaths over the last three decades. Part of this reduction is due to reduced fertility, and part is due to child-survival interventions.

The UN projection would require an average annual growth rate of 1.29% from today to 2050. As the CDR will not fall below 8/1,000, the CBR would have to remain, on average, at least 21/1,000 [The population growth rate (natural increase, not including international migration) is the difference between CBR and CDR, expressed as a percentage, not per 1,000]. This is unlikely as even if the TFR remains at a plateau at replacement level through 2050, the population-ageing process will bring the CBR down below 20 within a decade or so. Simultaneously, the population-ageing process will start to push the CDR beyond 8/1,000 from around 2020, which will further reduce the population growth rate.

### Replacement fertility

It is worth noting that the exact year in which replacement fertility is achieved is not of great importance. What is potentially of much greater importance is whether or not fertility can be brought below replacement fertility. For example, if fertility stops declining on attaining replacement level, the final population will continue to increase for another century and add at least 100 million. However, if fertility declined through replacement (i.e. TFR around 2.2) to the current level of Thailand (TFR around 1.8), the final population could stop growing at 205-210 million, some 50-55 million lower than otherwise will be the case.

### Age structure and population momentum

A demographic change that has major implications is the rapid fertility decline of the 1980s. The first impact has been to slow population growth, but it has resulted in a gradual change in age structure. An encouraging feature of the change to, or ‘ageing’ of the age structure, is the fact that the number of children and young people aged less then 15 years—now at 47 million—will never increase. Throughout this century, that number will remain stable, which should assist planners in building an education system.

A related feature of this age structure is that, with current low mortality rates, many of those 47 million young people will survive and pass through the reproductive years and contribute to the continuing population growth, even if replacement fertility level is attained within a decade [Every 5-year delay in achieving replacement fertility adds 3% to the population projection]. To illustrate the significance of this, there are currently about 45 million young women in the reproductive ages (15-49 years). By mid-century, this number will have increased to 75 million, as over 90% of young people who survive to age 15 years will survive to age 50 years.

### Determinants of future population growth

Can changes in any other determinants of population growth play an important role in what future population growth will be in Bangladesh? The major components include fertility, mortality, and migration.

#### Fertility

Following a dramatic and precipitous decline in the 1980s from almost seven children per woman (late 1970s) to just over three children per woman (early 1990s), fertility has been stagnant for a decade. Demographers found it difficult to predict when a decline would restart. The usual indicators of future behaviour, such as reported ideal family size and unwanted fertility, did not indicate a very substantial unmet need for fertility control; nor did the gradual decrease in gender preference, which still suggested a desire for at least one boy and one girl, which tends to result in an average fertility (TFR) of around three children, even if 70% of parents say that they only want one of each.

**Fig. 2:**
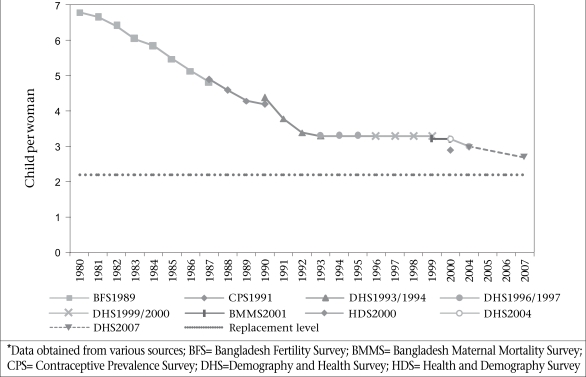
Trends in fertility, Bangladesh, 1980–2007

The only indication of a coming restart of fertility decline was the continuing fall through the 1990s in the less widely-used parity progression ratios. Indeed, the Demography and Health Survey (DHS) 2004 showed a minor drop in TFR to 3.0, which was of borderline significance [The TFR from BDHS 1999/2000 was 3.308 (3.159-3.457), and from BDHS 2004, it was 3.028 (2.894-3.161), meaning the 95% confidence interval barely touched at 3.16]. The latest DHS, in 2007, has confirmed the resumption of the decline (Fig. 2), with a further fall to 2.7, still half a child above replacement fertility. This author suspects that fertility will continue to fall gradually to a TFR around 2.5, then possibly a plateau again for some time, as some other countries have seen. There is still reason to be hopeful that replacement fertility will be achieved by 2015, as was the target for the 1994 World Bank population projections, although five years later than the 1996 UN PD projections.

### Family planning

A puzzling feature of the fertility plateau was the fact that contraceptive prevalence rates (CPRs) continued to rise steadily throughout the 1990s, from 44.6% (1993/1994) to a peak of 58.1% in 2004, followed by a slight fall to 55.8% in 2007, due to a fall in traditional methods. Experience from other countries suggests that such a rise in the CPR would produce a concurrent decline in fertility. Recent studies have not satisfactorily explained this paradox. An examination of the range of fertility determinants indicates that potentially important factors, such as delayed age at marriage, do not appear to have played a counter-balancing role in the Bangladesh case; nor have less powerful factors, such as postpartum amenorrhoea linked to breastfeeding, changed in any significant way. There has been a gradual shift in the use of permanent and long-term contraception towards a greater dependence on temporary methods, but this is mainly among older fertile women who are not contribu- ting much to overall childbearing rates. One possible factor may be that adoption of modern contraception has gradually been substituting for reliance on induced abortion. This pattern, seen in Matlab (2,3), appears to require quite high levels of CPR before a substitution begins to take place. This may well be context-specific and presumably depends on the level of fertility and the speed of the decline.

### Are new indicators needed to monitor child survival

Traditional measures of child mortality use, as their denominator, the population ‘at risk’, that is the numbers of children born. Defining the ‘at-risk’ population is central to epidemiological concepts; however, it also assumes that live infants within the cohort are uniformly at risk. Many confounders, such as age of parents, socioeconomic status, parity, and education of parents, alter this risk and, thus, affect the IMR. Since the acceptance of fami-ly-planning methods may be highly dependent on these same factors, the use of the IMR when used as a single indicator is not able to assess the interaction of these factors. There are other indicators of infant mortality, termed fertility-adjusted infant mortality ratio (FIMR), age-specific, fertility-adjusted IMR (AFIMR), and total infant mortality ratio (TIMR) which are more sensitive to rapid demographic changes and which take into account the combined effects of changes in both fertility and infant mortality rates on overall infant mortality in an entire region (4). These new indicators appear to better measure the effects of an integrated health programme. For the Matlab field area, the reduction in the numbers of infant deaths, as reflected by these indicators, is much greater than indicated by the IMR. Also, the new indicators conceptualize the mother-infant pair as an appropriate unit with which to monitor mortality and may be used for guiding allocation of resources intended to lower infant mortality and may better reflect the perception in infant mortality status in the community.

### Age at marriage

In a country undergoing a major fertility transition, it is usual to see a gradual delay in age at marriage for young women. Bangladesh is unusual in that the traditional pattern of very early marriage and high fertility has been transformed with virtually no evidence of marriage being delayed. One important indicator of early marriage is the proportion of teenage women (aged 15-19 years) currently married. Bangladesh has, until recently, maintained one of the highest proportions—close to half—of teenage marriage in the world, matched only with a few West African countries.

What is surprising about this unchanging early-marriage trend is that substantial investments have been made in the past decade to increase access by girls to primary and secondary education. Free primary schooling for girls to grade 12 (with plans to extend it to higher level) and a national female secondary school stipend scheme (FSSSS) which provides financial support to parents who keep their daughters in high school as long as possible, with the provision that the girl does not marry [There are other eligibility criteria, such as maintaining a minimum pass rate (45% or so)]. There is a draft policy proposing to extend this scheme to girls from poor families in urban areas. Thus far, there is little evidence of these educational inputs translating into delayed marriage, as the historical experience from other countries would predict.

It is probably safe to predict that continued investments in female education will result in delays in early marriage in the near future—at least among some groups. This, in turn, will contribute to further fertility decline. What is less certain about mass education schemes, such as the FSSSS, is whether girls from poor families, who now achieve high levels of education through such schemes, will behave similarly to girls who historically had high education and tended to come from economically well-off families with different knowledge, experience, and values. There easily could be a delay in observing later marriage among poor families.

### Ageing

**Fig. 3:**
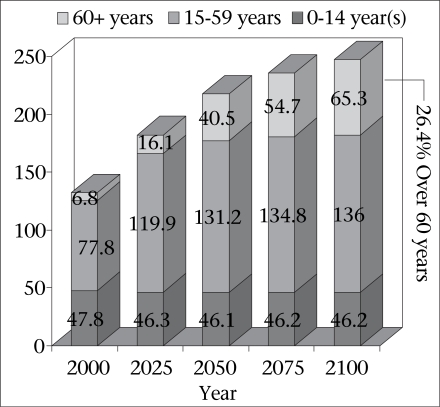
Bangladesh population (millions) in 21st century

Another less-widely discussed aspect of the ‘ageing’ of the population is the impending massive growth of the elderly sector, aged 60 years and over, during this century. Typical of most developing countries, the elderly of Bangladesh currently constitute only about one in 20 of the population. However, during this century, the current seven million will grow to 65 million, by then accounting for some 26% of the total population (Fig. 3). This ‘ageing’ shift has major implications for health as more than half of all deaths occur in this age range.

It is partly this ageing trend that is triggering the shift from infectious to non-infectious diseases as the major causes of death. It is anticipated that, by 2010, non-communicable diseases will account for 59% of all deaths, up from 40% in 1990. The proportion due to communicable diseases will drop from 51% to 30% over the same period. The remainder is due to injuries, which will increase a little, mainly as a result of more road accidents.

It can safely be assumed that, during this century, a health service system, probably incorporating some form of health insurance, will evolve in response to these new demands. Is this a safe assumption? Who is going to pay for these services, which will inevitably be much more expensive than existing services for most infectious diseases? While the elderly population grows at a rapid rate (2.2% per annum), the working-age population will grow much more slowly, only 0.5% per annum. The effect of this will be that the present dependency ratio will fall precipitously from 11 workers per one elderly person to three workers by 2050 and to two workers by 2100—similar to current Western ratios. Even assuming an effective taxation system in place, the burden on workers to pay for health services will be greatly increased. In the next section, the implications of ageing for health services will be briefly discussed.

### Implications of population growth and ageing for health systems

There will be two ways in which population growth impacts patterns of disease and the need for health services. One is simply the increase in numbers, around 38% over the coming three decades. The other is the impact of the changing age structure. A simple exercise will illustrate these two effects. Applying the WHO GBD age-specific prevalence rates for diabetes for South Asia to the growing Bangladesh population, then applying costs per case of treatment (at current retail prices and assuming all cases receive complete treatment), suggest that the number of cases will rise from 2.7 million in 2005 to 7.1 million in 2035. The implied costs will rise from US$ 154 million to US$ 405 million (@US$ 57 per case). This is a 162% increase in health costs, of which only one-quarter is due to growth in overall numbers, plus three-quarters due to ‘ageing’ of the population.

The impact of ageing is not limited to non-communicable diseases, but infectious diseases that afflict adults, such as tuberculosis (TB), are also important. Using the same approach, the costs of complete TB treatment for all cases would rise by 76% from US$ 33.7 million to US$ 59.6 million, of which half the impact would be due to the increase in numbers of population and half due to the changing age structure.

The impact of ageing is much greater with diabetes than TB because the age-specific rates rise much more steeply with diabetes, and it is not presently a curable disease, so the duration needed for management is much longer.

While the rapidly-rising number of cases of both non-communicable and communicable diseases of adults has major cost implications for the health system, it also suggests that issues relating to skill balances and medical school curriculum need to take account of the changing demands.

## GEOGRAPHIC DISTRIBUTION OF THE POPULATION AND URBANIZATION

Not only are the total population figures important, their distribution between urban and rural areas is also critical. Overall growth may continue through the present century, but the rural population will stop growing by 2025, adding ‘only’ another 30 million to the current 108 million. The urban population, however, will absorb the additional 70 million national population growth, so the current urban population of 34 million will blow out to over 100 million, possibly during this century. This frightening growth will be driven predominantly by rural-urban migration, with a small component of natural increase.

How can urban populations grow at this rate, tripling in size within this century? The current rate of growth of the urban areas within the six metropolitan areas is around 3.5% per annum. The BBS quotes a rate of natural increase for urban Bangladesh of about 1.3% per annum. If this estimate is correct, the difference (2.2% per annum) must come from rural-urban migration. This gives a doubling time of 20 years [The doubling time is estimated by dividing the annual growth rate in percentage into 69.3, or 70. Populations grow exponentially, and the natural log of 2 is 0.693], or a tripling time is about 31 years. So, a doubling is probable by mid-century at the current growth rate. More important even than the overall urban growth rate is that the slum populations within the urban areas are growing at double the average urban rate—around 7% per annum. For example, in Dhaka, more than one-third (37.4%) of the 9,136,182 population in the metropolitan area currently live in slums. Nationwide, that proportion is similar, at 35.2%, although in other divisions the proportions are lower (Khulna City Corporation=19.5%). The growth rate for urban Dhaka indicates that, in absolute terms, it is growing at around 320,000 persons per annum in the metropolitan area, of which three-quarters are added to the slum population. This proportion is likely to be similar elsewhere, although the total numbers will, of course, be smaller than in Dhaka.

**Fig. 4:**
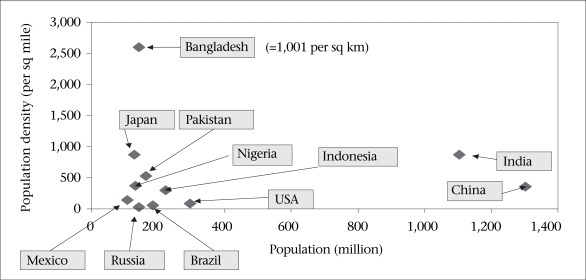
‘Mega’ countries with population of >100 million

Figure 4, showing Bangladesh with a current population density of 2,600 per sq mile, supports the argument that Bangladesh is a special case in that its economy is very much based on agriculture, but its exceptional population density places it at great risk of reaching saturation in terms of agricultural production and capacity to absorb further population increases into the rural labour force [To place the Bangladesh population density in perspective, if the entire world population was squeezed into the USA, the density would be 1,740, or into Australia, the density would be 2,170 per sq mile; The population density of Bangladesh will rise from 2,700 to 4,500 per sq mile by 2050].

There is a slightly encouraging sign that urban growth appears to be slowing in recent years. While there is little evidence, this is possibly linked to the rapidly-expanding microcredit facilities in rural areas, and consequent employment and income-generation taking some pressure off the poor to move to the cities in search of work.

### Urban health conditions and challenges

The health of the urban poor or slum dwellers is primarily due to crowding and lack of access to basic services, such as water and sanitation. Consequences of these living conditions include stress due to crowding, insecurity due to lack of housing and land tenure, various types of illegal or criminal activities, including violence, drug-use, prostitution, etc.

If conditions are so bad, why do people move to these areas? Presumably because there are better (perceived) chances to find cash from employment.

### Population density and crowding

**Fig. 5:**
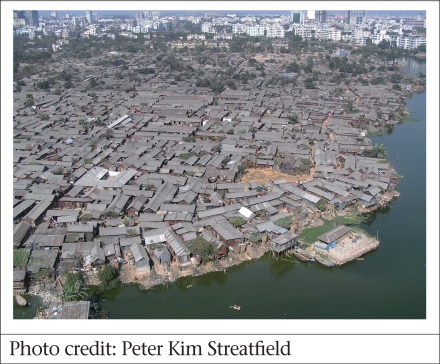
Korail slum in Dhaka

Rural Bangladesh has a relatively high population density of 755 per sq km This is sparse compared to the urban population densities from the latest slum-mapping (5), which are 30 times higher, averaging 23,378 nationwide (7,152 in Barisal City Corporation to 29,857 in Dhaka metropolitan). The slums, however, at 205,415 persons per sq km, are almost 300 times more densely populated than the rural averages. Figure Fig. 5 illustrates the point that most slum housing is single-story, not high-rise and, thus, very crowded. Chittagong is the highest at 255,100 persons per sq km. With modal (most common) values of 4-5 persons per room and the average house-size of 103 sq ft, there can be little doubt that crowding is a genuine issue.

Since slums tend to be in low-lying areas, where the better-off do not want to live, they are subject to flooding (26.5% fully flooded, 27.4% partly flooded, 46.1% flood-free), with over half of the area being poorly drained (52.7%, plus 37.0% moderately drained). More than half (55.7%) have no fixed place for the disposal of garbage. More than half (52.8%) of the population use a pit latrine for sanitation, and in many households, these must be shared—in one in eight slums, one latrine was shared, on average, by 11 or more families.

What are the prospects for this situation with future rapid urban growth? Water is likely to be a limiting factor. At present, the people of Dhaka city need 2.1 billion litre of water a day (L/day). Dhaka WASA provides 1.45 billion L/day, mostly from groundwater through its 400 deep tubewells, and the remainder is from the Shitalakshya River, which is becoming increasingly polluted. Many additional deep tubewells are serving the mushrooming numbers of high-rise buildings, and the overall impact of extraction of this underground water at a rate exceeding the capacity to recharge the aquifer is a drawdown of the Dhaka water table at an unsustainable rate. The water table is presently over 60 metres below the surface [One positive aspect of this low water table is that it is below the level of arsenic contamination of the shallow aquifer, down to 30 metres], some 20 metres lower than eight years ago, and 35 metres lower than in the mid-1970s. Surface water should be a viable alternative [There is a proposal to import relatively clean surface water from areas to the south-west of Dhaka city, as the Shitalakshya river has reached its extraction limit due to pollution and siltation]. However, surface water is contaminated by industrial wastes (mostly toxic chemical dyes, heavy metals, etc.) and domestic wastes due to the inadequate sewerage system.

Only Chittagong and Dhaka are the two cities with any piped, water-based sewerage system, but it is very old and poorly maintained. Only 30% of city residents live in areas served by the sewerage system, another 30% have septic tanks, and the rest lack even basic sanitation, further exacerbating the pollution of surface water.

While the recommended allowance of water is around 200 L/day per person (for all purposes), many slum-dwellers manage with less than 10 L/day. With the Dhaka population increasing by over 300,000 persons each year, theoretically needing six million additional litre per day, the numbers limited to this inadequate amount or less, will undoubtedly increase, unless something drastic is done.

The health implications must be clear, where populations are growing, water is becoming increasingly scarce, and sanitation is poor and not improving, waterborne diseases are very likely to become a serious problem again.

In addition, air-borne diseases, such as influenza, pneumonia, and TB, which tend to be exacerbated by crowding, are obviously also likely to increase under these pressured conditions.

## CARRYING CAPACITY OF THE BANGLADESH POPULATION

There can be no doubt that the continued growth of the large population of Bangladesh has major implications in the future development of the country. Questions that are not often asked are what population can Bangladesh support; what strategies might be used for minimizing the negative impact of future population growth; and what changes to population distribution and the economy will be needed.

There are several issues that throw light on this. What is the capacity of agriculture to generate employment and absorb the expanding population into gainful employment? What is the capacity of agriculture to feed the growing population?

Bangladesh has 8.774 million hectares of cultivable land, of which 88% is cultivated, so there is a limi-ted scope to expand the cultivated area. Intensive double (59%) and triple (22%) cropping increases the effective crop production by 150%. Has Bangladesh reached the limits of agriculture? This may depend in the short term more than anything else on the availability of water [In the longer-term, the shift to genetically-engineered or modified crops might play a role in Bangladesh, just as the introduction of high-yielding rice varieties did in the 1970s and 1980s].

Bangladesh resides in a water paradox in that it receives massive volumes of water both from transboundary rivers and from monsoon rains (average 2.3 metres annually), but water shortages are common. Among the 57 transboundary rivers are the Ganges [The Ganges carries so much water into Bangladesh that the Paksey bridge near the Indian border needs the deepest pylons in the world—91 metres below the riverbed], the Brahmaputra, and the Megna, with peak discharges among the highest in the world according to the Food and Agriculture Organization (6). Between these and other minor rivers, some 1,100 km^3^ of water enters Bangladesh annually, mostly during the monsoon (June-October). To put this in perspective, it is equivalent to covering the entire country in water to a depth of 7.5 metres.

How can there be water shortages? There are two reasons. The first is that, across the 230 rivers, dams capture less than 2% of internal renewable water resources. Irrigation is used on only half the potentially-irrigable land area, and major canals cover less than 10% of the total irrigated area. The remaining area uses minor irrigation systems, with pumps for groundwater, shallow and deep wells of various kinds. However, a concern is that “there has been a general reduction in the area irrigated by wells as a consequence of aquifer drawdown, and there has been an increase in salinity, particularly along the coastal areas in the southwest of the country” (6).

The second reason is that, in the dry season, the groundwater reserve amounts to less than 2% of total renewable water resources, yet due to lack of dams and canals, this forms a major source of irrigation water. When irrigation is most needed, farmers suffer water shortages due to erratic power supply, which limits their capacity to operate electric pumps to access the shallow and deep aquifers. Yet, despite this ‘dry-season drought’ situation, over the past four decades, about half of the funds spent on water development has been for embankments for flood control and drainage projects. A further consequence of these embankment projects has been a gradual reduction in the fertility of the soil due to preventing the natural siltation associated with flooding. Farmers living within embankments may be able to multiple crops—rice and other cereals, but they report needing increasing amounts of artificial fertilizers each season. It is not only embankments which affect crop production, but “man-made soil degradation is mainly due to continuous rice monoculture, inadequate soil conservation measures, and unbalanced fertilizer use” (7).

Unless major changes are made in access to water sources for irrigation in the dry season, agricultural production will not increase significantly. The optimistic view is that food production will keep pace with the growing population at least until 2010, when requirements of food-grains will be 31 million metric tonnes (mt), and production will be about 30 million mt. These national averages may conceal household-level variations. For example, assuming a daily per-capita requirement of 454 g of food-grains, an average family of five would need 825 kg annually. With average agricultural land-holding at 0.2 acres and average production at 4 mt per acre, the typical agricultural family can currently produce almost exactly their intake requirements of 0.8 mt (800 kg). However, this leaves no excess for sale and for supporting the population in non-agricultural households in the country.

The National Water Management Plan may contribute to changes in patterns of water extraction that may alleviate some of this problem, but if agricultural production does not increase, what will be the trend for agricultural employment?

The latest figures suggest that, compared to the mid-1990s, the numbers working in agriculture are about 17.2 million males and 5.8 million females and are stagnating or declining. Figures on small or zero land-holdings by farming households are elusive. The 1996 agricultural census indicates that 10.2% of farm households owned no land at all, followed by 79.87% holding between 0.05 and 2.49 acres (0.02-1 ha) (8). This range is not useful as it covers impossibly small areas to reasonably large areas, but includes most farm households. What is clear is that farms in all size categories have declined between the 1983–1984 and the 1996 agricultural census, and the overall average farm size has fallen by one-quarter from 2.26 acres to 1.69 acres (8). Measures of landlessness are complicated, but it appears that, from the early 1980s to the mid-1990s, landlessness fell from 56% to 50%; however, micro-level evidence suggests that the “rate might have slowed down in recent times in part as a consequence of the escalation in the pace or rural-urban migration” (7).

These trends suggest that agriculture will not absorb the continuing growth of population into the economically-productive labour force. Thus, the continuing short-term growth in the rural areas of around two million annually will have to find other sources of employment, of which there are a few, or they will be driven into the cities looking for work. The latter is the most likely scenario. By 2025, when rural populations are expected to stop growing, migration to urban areas will then balance the annual natural increase of 1.3 million.

It is worthwhile to point out that the area of cultivable land is not static. It is constantly under threat from the need for land for other uses. Between 1983–1984 and 1997, the area of cultivable crop-land declined by almost 14% while the area for homestead increased by 20%; adjacent ponds and other residential uses increased by another 20%, consuming over 300,000 acres of crop-land. The growth of district-level small cities and towns is now having a noticeable impact: “urbanization offers the greatest substantive threat to total land available for agricultural use” (7).

Another aspect of the ‘water paradox’ is that global trends in climate change pose threats of rising sea-levels which may inundate coastal areas and further reduce available agricultural land by both direct flooding and salination of the aquifer, as is already occurring in Barisal. This prospect is discussed further below.

Finally, in this brief overview, the potential catastrophic impact of the proposed plans to divert many major rivers passing through India will not be discussed. Obviously, if this had occurred, Bangladesh would be deprived of currently-available transboundary surface water. The only positive aspect of such a scheme might be a reduction in numbers of people rendered homeless by the perennial problem of river erosion.

River erosion is believed to swallow nearly 25,000 acres (10,000 ha) each year, leaving some 60,000 people homeless. It is predicted to consume 3,575 sq km by 2025. The Brahmaputra and Jamuna rivers alone are said to have contributed to one million rural inhabitants being driven into poverty, and forced to relocate, with the trend in recent decades of moving to the cities in search of work. According to Oxfam, up to 90% of trafficked women and children are victims of river erosion, forced to migrate from their rural homes.

## CLIMATE CHANGE AND ITS IMPACT ON BANGLADESH

There is a growing recognition that climate change in terms of global warming precipitating rising sea-levels is a genuine harmful phenomenon and is a potential threat to low-lying countries, such as Bangladesh. Surprisingly, there appears to have been a few simulation exercises to try to predict how many people and how much land would be affected by rising sea-levels. While accepting that the rate of climate change will be determined by many factors, and by circumstances already in place, the simulations by University of Middlesex, UK, suggest that South Asia will be massively affected, with up to 55 million people affected by flooding, if there is no change in their present levels of CO_2_ emissions and atmospheric warming (9). This impact could be reduced to 20 million if CO_2_ emissions are stabilized at 750 ppm [Pre-industriali-zation levels averaged around 280 ppm], and to 10 million if reduced to 550 ppm.

Two qualifications here are that many of those affected in South Asia will be on the Indian coast, but Bangladesh currently has around 30 million people living in vulnerable coastal areas and islands in the Bay of Bengal. The second qualification is that the time scale is believed to be relatively long in political, if not geological, terms. The Hadley Centre for Climate Prediction and Research estimates that sea-levels will rise about 40 cm (15 inches) by 2080.

**Fig. 6:**
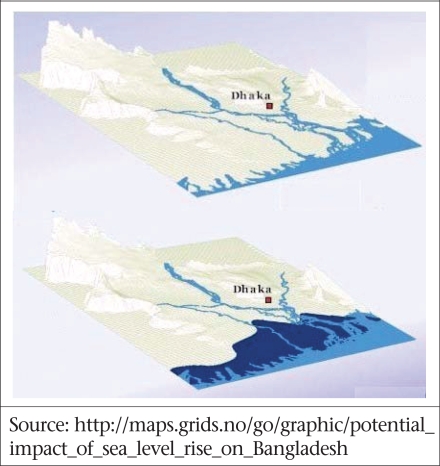
Add a caption and legend

It is frustratingly difficult to find suitable topographic maps to allow estimates of the current population potentially affected by a certain rise in sea-level. An alternative and more pessimistic prediction by GRID Arendal (Fig. 6) is that sea-level might rise 1.5 metres by 2030, in which case 22,000 sq km (16%) of land area would be affected, equivalent to some 34 million population. This latter estimate is quite dated by now, and new estimates are needed. The SURVAS project (www.survas.mdx.ac.uk) is in the process of updating some of these simulations for the developing world (9).

A recent study has suggested a catastrophic possibility of sea-levels rising by as much as six metres by 2100 due to more rapid-than-expected melting of the polar ice-caps (10). Such an event would swamp vast areas of Bangladesh and cause extreme hardship and damage to agriculture and other crops, e.g. pond-based shrimp production, even if there was time to warn and relocate coastal residents (See current and projected coast lines of Bangladesh.)

## CONCLUSIONS

Bangladesh has achieved considerable success in reducing fertility but population momentum will carry its population close to 250 million, unless something very dramatic and unforeseen occurs to bring fertility below replacement level within a decade or two.

Future trends in fertility will depend on further improvements in child survival, which has been stagnant for the past decade, following impressive gains in the 1980s. It will also depend on the changing status of women and girls, and that will require continued inputs in primary and secondary female education linked with developing formal sector employment opportunities in the rural areas, not only in the cities.

A key demographic shift that demands a response is the rapid ageing of the population due largely to the fertility decline. Numbers of elderly people will increase six-fold by mid-century, while numbers in the working ages will less than double. This decrease in the relative size of the working age tax base to the growing demands for healthcare by the elderly group, who are more prone to non-communicable diseases, should be a matter of concern, as Bangladesh evolves health insurance schemes, and more expensive health services, in response to changing needs.

Even simplistic exercises on the burden of diseases can demonstrate how ageing will bring a major shift to non-communicable diseases, with consequent change in demand for more expensive and more sophisticated health services.

A second demographic phenomenon that demands an immediate response is that of rural-urban migration. The above review of the future of rural agriculture suggests that Bangladesh is close to the limits of availability of agricultural land and productivity, at least in the short-term. This is, partly, due to challenging problems of satisfactorily controlling the flow and timing of water for irrigation and, partly, due simply to the very high population densities in the rural areas.

The inevitable consequence of this situation will be urbanization on a scale which risks overwhelming the capacity of the urban authorities to provide housing, water and sanitation, healthcare, education, and other essential services to incoming migrants. A recent study and mapping of slums indicates that they are growing at over 7% per annum, implying a doubling time of less than a decade. This frightening scenario requires an immediate strengthening of urban planning and consideration of how to slow the pace of movement away from rural areas.

### How are these challenges consistent with the MDGs?

The following list shows the targets within the MDGs that are potentially affected by population growth. In some cases, the effect is more direct than in others.

#### MDG 1—Target 1: Halve the proportion of poor people

Fertility is 60% higher among the poorest quintile than among the least poor. However, the poorest group is growing faster, and the child survival is lower.

#### MDG 1—Target 2: Halve the proportion of hungry people

Food production has kept pace with population growth for the past few decades, but may not continue due to reaching the limits of domestic agricultural production.

#### MDG 2—Target 3: Ensure that boys and girls can complete primary schooling

The number of children eligible for primary schooling has now stabilized and will not increase further. This should facilitate planning and hopefully implementation.

#### MDG 4—Target 5: Reduce under-five mortality rates

There is evidence that the fertility decline has reduced numbers of childhood deaths, if not rates. It has reduced selected high-risk pregnancies. Some risks, such as drowning, may have increased due to reduced toddler supervision.

#### MDG 5—Target 6: Reduce the maternal mortality ratio

The fertility decline has reduced selected high-risk pregnancies and the absolute numbers of maternal deaths considerably. There is potential for further reductions, if early childbearing can be reduced by delaying marriage.

#### MDG 6—Target 7: Reverse the spread of HIV/AIDS

Factors accelerating rural-urban migration contribute to changing family structures where young women and young men are exposed to risks of urban life. These exposures were not found in traditional Bangladeshi life. The poverty-driven search for employment can result in circumstances where high-risk behaviours become acceptable.

#### MDG 6—Target 8: Reverse the incidence of malaria and TB

Rising population densities can force people to migrate to malaria-endemic rural areas, thus adding to the populations at risk of resistant *Plasmodium falciparum* malaria. High-density living and crowding is a risk factor for air-borne diseases, such as TB. Other infectious diseases, e.g. pneumonia and diarrhoeal diseases, are likely to increase due to crowding and unsanitary conditions. Global warming will likely increase the risk for certain vectorborne diseases, e.g. dengue.

#### MDG 7—Target 10: Access to safe water and basic sanitation

As described, rapid urbanization will create possibly insurmountable pressures on safe water supplies, and the insecure tenure of slum-life, tends to be associated with lack of basic services, as governments are reluctant to provide them even if resources are available.

#### MDG 7—Target 11: Improve the lives of slum-dwellers

As above, governments historically tend to avoid providing services to slum-dwellers in fear that it will simply pull more rural migrants into the cities.

#### MDG 8—Target 16: Strategies for decent and productive work for youth

In a slowly-modernizing economy, the rapid growth in numbers of youths seeking employment can exceed the capacity of the private and public sectors to generate jobs.

#### MDG 8—Target 17: Provide access to affordable, essential drugs

The steady shift towards non-communicable diseases resulting from ageing of the population—a direct result of the fertility decline—will carry major implications for the types and costs of drugs and health services for managing common health problems.

### THE WAY FORWARD

***Fertility:*** Evidence suggests that the recent cessation of outreach family-planning services has selectively disadvantaged the poor, among whom fertility has risen. If this is true, the importance of services for different population subgroups need to be better understood. The same pattern may also apply to women in Sylhet, who may be economically better-off, but have limited mobility. Their fertility has been consistently higher than in other parts of the country. Learning how to develop health systems to identify high-fertility families and target these families for additional services will be a challenge to the health system.

**The way forward**

- Targeting family-planning programmes to high-parity families and those at high risk of unintended pregnancies.
- Emphasizing longer-term methods for family planning.
- Preparing for urbanization—improving urban infrastructure and a skilled workforce.
- Prevent and control diseases of urban slums: tuberculosis, enteric infections, pneumonia, and violence.
- Prepare for and provide health services for urban areas.

***Family planning:*** Bangladesh still has a pattern in the use of short-term family-planning methods which are rather inefficient where most women marry while young and complete their childbearing in their mid-twenties, leaving another 20 years of fertile life to ensure protection from unwanted pregnancies. It is possible that (justifiable) concerns about poor quality of care and fear of surgical procedures underlie much of the hesitation to adopt long-term or permanent contraceptive methods. Much has been said about this, but little improvement has eventuated. What can be done about this?

***Rural-urban migration/urbanization:*** Does the provision of microcredit used for income and employment generation slow the process of rural-urban migration? Many rural people migrate to urban areas with the hope of finding employment. In many cases, this hope remains unfulfilled. What is the nature of employment in the urban informal sector, and how can employment opportunities be increased in urban areas? Is there a role for urban microcredit? Can it be linked to other direct health interventions?

***Water and sanitation:*** What can be done to improve water and sanitation and to minimize water-borne diseases in slum conditions? How much is social mobilization effective in creating groups who can organize, for example, funds to sink functional deep tubewells? Or use community savings to construct safe, hygienic latrines?

There are numerous examples, from neighbouring countries, of group formation to alleviate the pollution of surface water supplies for urban dwellers. This tends to involve blocking raw sewerage outlets into city-lakes, preventing industrial wastes from entering city-lakes and ponds, and exposing and preventing further unauthorized land-grabbing of wetlands in urban zones. In addition to expanding the supply of available water for domestic use, it can reduce monsoon flooding in the vulnerable low-lying slums.

***Drug use, violence, and illegal behaviours:*** The HIV/AIDS epidemic is likely to start among drug users in Bangladesh. They are not uniquely urban, but urban conditions tend to be associated with this and other illegal behaviours. What underlies this tendency? What can be done to minimize it and to redirect young people into more meaningful and productive activities?
